# Supplementary material for: Schlafen 12 restricts HIV-1 latency reversal by a codon-usage dependent post-transcriptional block in CD4+ T cells
Source: Commun Biol. 2023 May 10;6:487. doi: 10.1038/s42003-023-04841-y (PMC10172343; doi:10.1038/s42003-023-04841-y)
Supplement: Supplementary file 2 — Supplementary Information [file 42003_2023_4841_MOESM2_ESM.pdf]

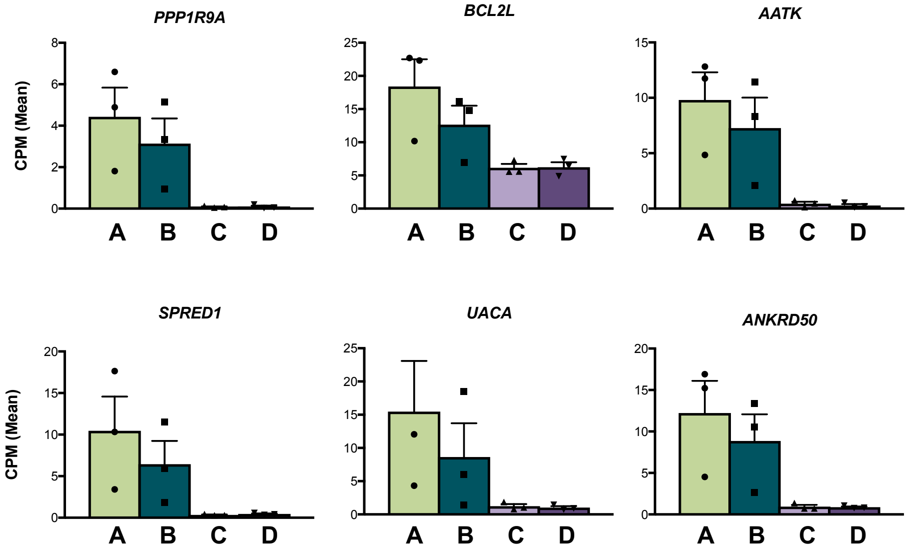

Supplementary Figure 1. Expression patterns of the rest six genes in the final candidate list.

Plots show expression levels of the candidate restriction factors, *PPP1R9A, BCL2L, AATK, SPRED1, UACA, and ANKRD50* obtained from RNA-seq (mean CPM ± SEM; n=3). The numerical source data is available from Supplementary Data 1.

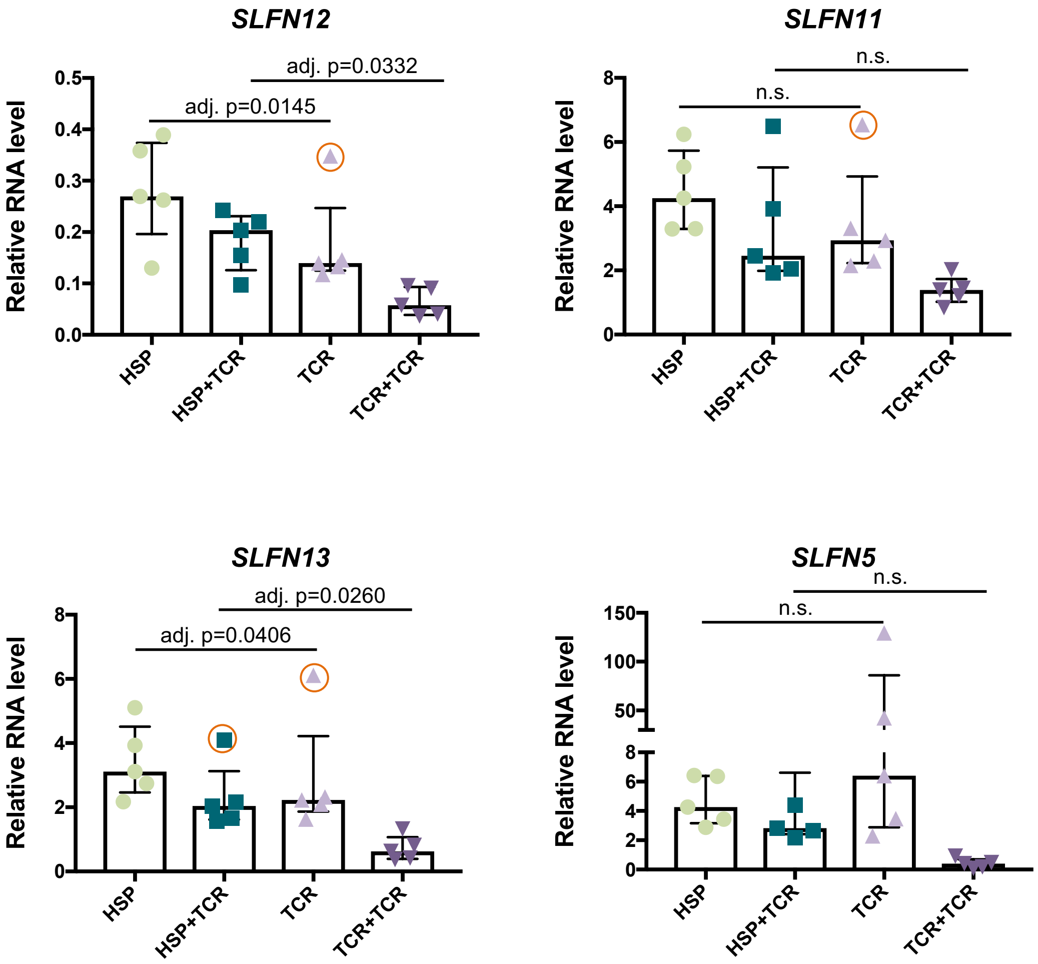

Supplementary Figure 2. RT-qPCR data of five additional healthy donors cultured with HSP or TCR conditions.

The bars show the median ± interquartile range (n=5). *SLFN14* was under the detectable limit in all the samples we tested. Outlier values (highlighted with orange circles) were identified by ROUT method[1]at maximum desired FDR (Q) =5% and removed to calculate adjusted p-values (adj. p) by one-way ANOVA per each gene. n.s., insignificant difference (adj. p > 0.05).

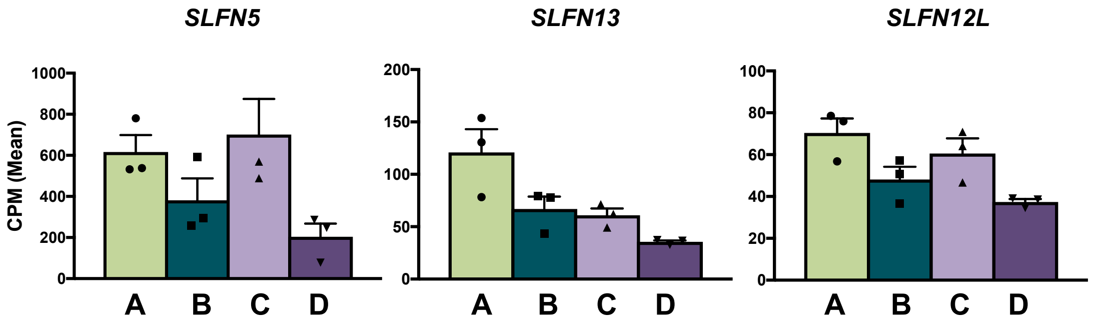

Supplementary Figure 3. Expression patterns of *SLFN* family genes *5, 13 and 12L*. Given are the plots of RNA-seq data (mean CPM ± SEM; n=3). *SLFN14* and *SLFNL1* showed no read count. *SLFN13* and *SLFN5* were classified into cluster II by the k-means clustering shown in Fig. 1c.
The numerical source data is available from Supplementary Data 1.

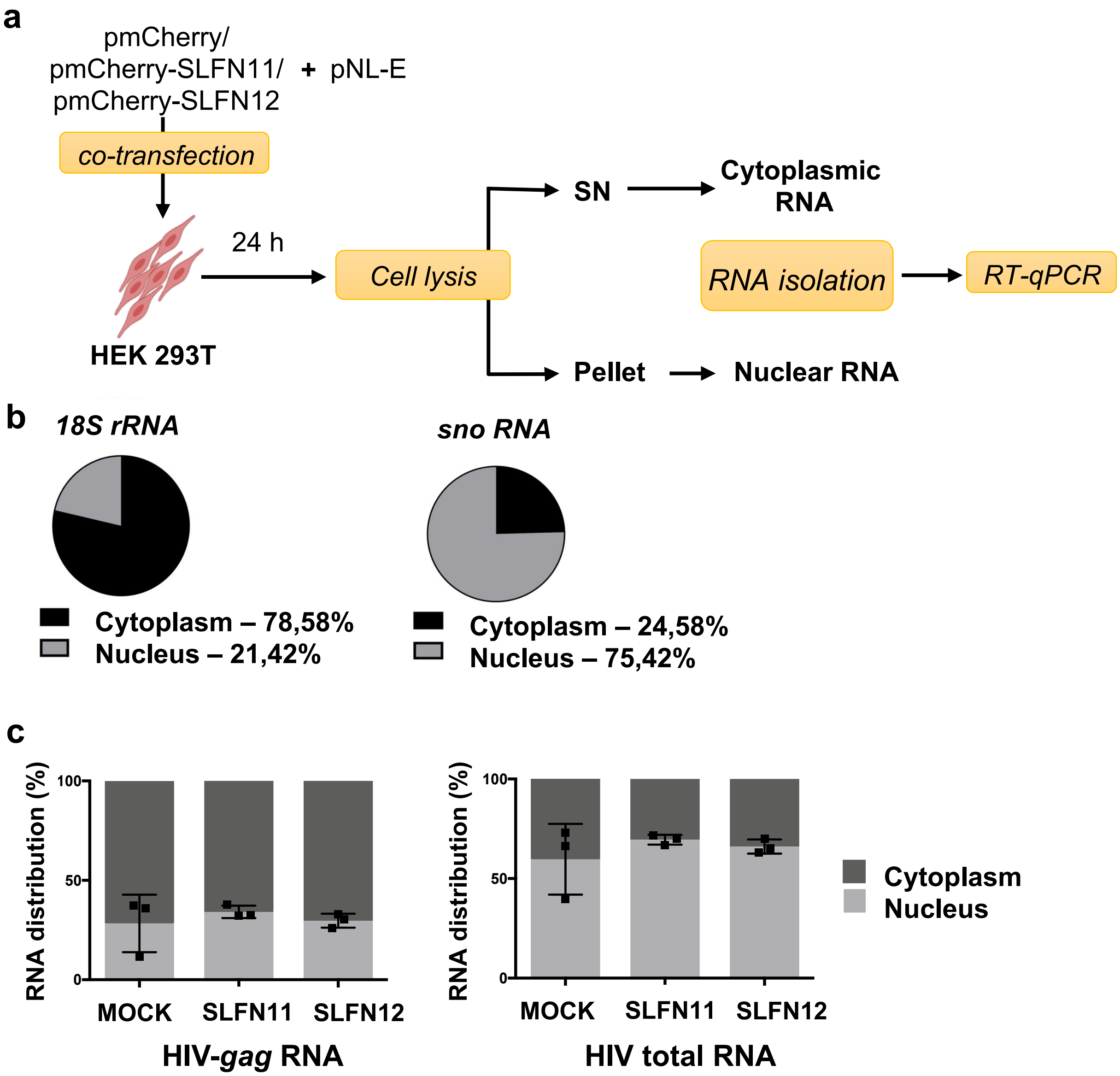

Supplementary Figure 4. SLFN12 does not affect HIV RNA distribution.
a Scheme of this experiment. HEK 293T cells were co-transfected with pmCherry-SLFN11/12 or pmCherry empty vector along with HIV pNL-E vector. 24 hrs post-transfection, the cells were lysed in a hypotonic buffer to separate cytoplasmic fraction (supernatant) and nuclear fraction (pellet). RNAs were extracted from each fraction and analyzed by RT-qPCR. **b** Fractionation efficiency. *18S rRNA* and *U3 small nucleolar RNA* (*snoRNA*) were quantified as controls for cytoplasmic and nuclear RNAs, respectively. Cytoplasmic and nuclear RNA distribution shown in percentage was calculated as an average of independent triplicates from mock-transfected HEK 293T cells. **c** Distribution of HIV-*gag* RNA (Left) and HIV total RNA (Right) in the transfected HEK 293T cells. The total amount of HIV-*gag* or –total RNA in both fractions (cytoplasmic and nuclear) was set to 100%. Error bars represent the standard deviations of three independent samples.

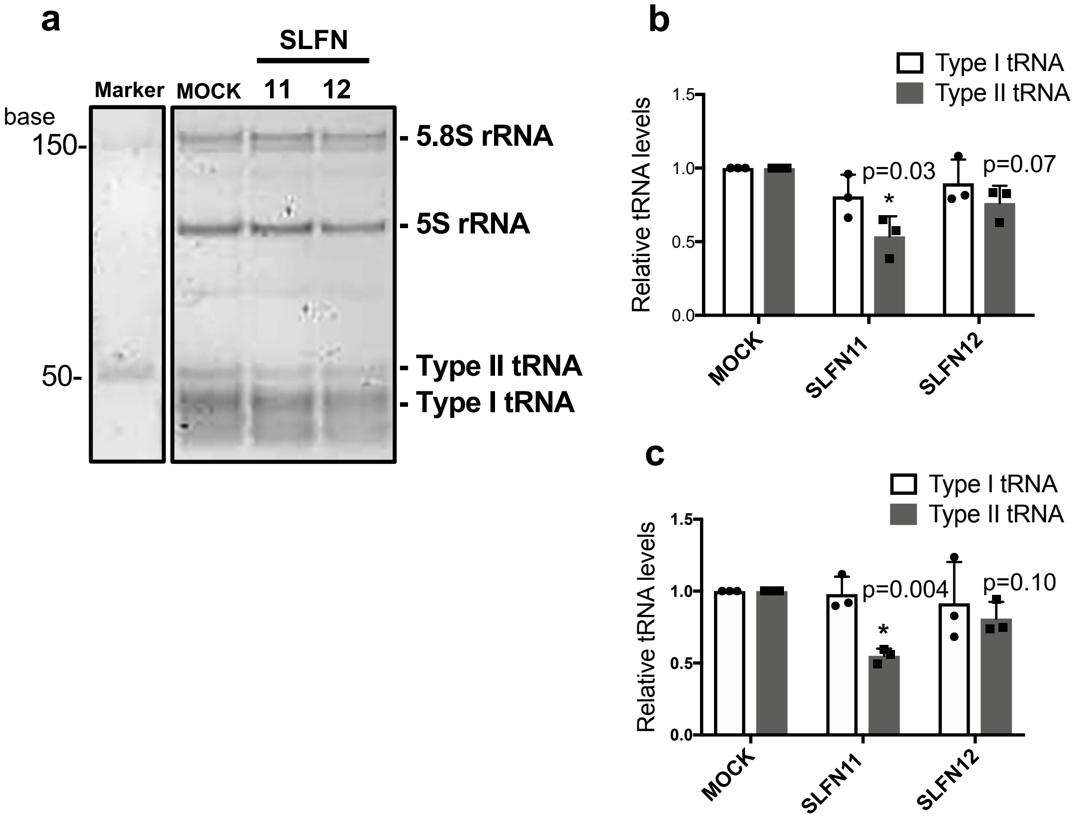

Supplementary Figure 5. tRNA levels in cells after SLFN11 or SLFN12 expression.
a Representative result of RNA electrophoresis. Total RNA extracted from mock/SLFN11/SLFN12-expression vector-transfected cells were resolved on a 10% denaturing 10M urea polyacrylamide gel. **b and c** Quantification of results from **a** (n=3, mean ± SD). Plots show intensities of the type -I and -II tRNA measured by Image J and normalized by 5.8S rRNA intensity (**b**) or 5S rRNA intensity (**c**). The levels in mock-transfected cells were set to 1. p-values were calculated by one-sample t-test.

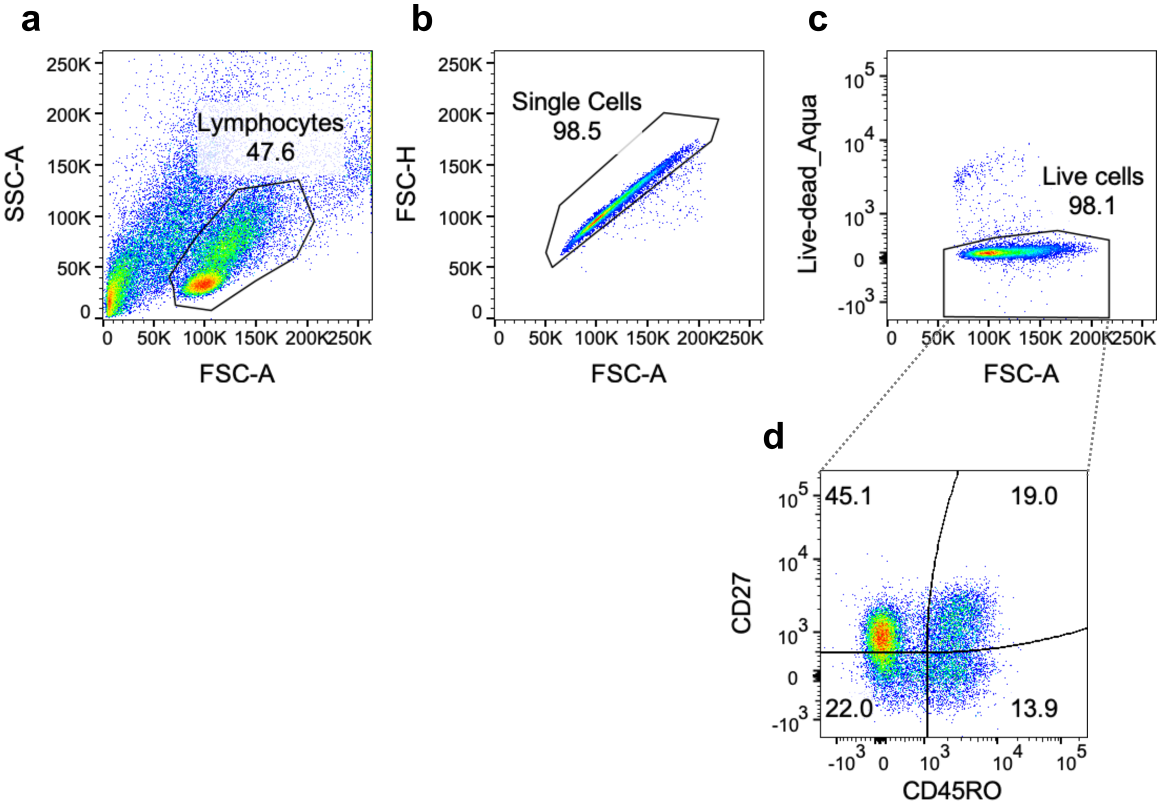

Supplementary Figure 6. Gating strategy of flow cytometory, related to Fig. 1b.
a-d The cultured naïve CD4+ T cells were gated into lymphocytes (**a**) by FSC- and SSC-scatter, followed by single cell selection (**b**) and dead cells exclusion (**c**), and finally analyzed intensities of CD27-Alexa Flour 700 and CD45RO-ECD (**d**). The figure was illustrated with HSP-cultured naïve CD4+ T cells obtained from donor#2.

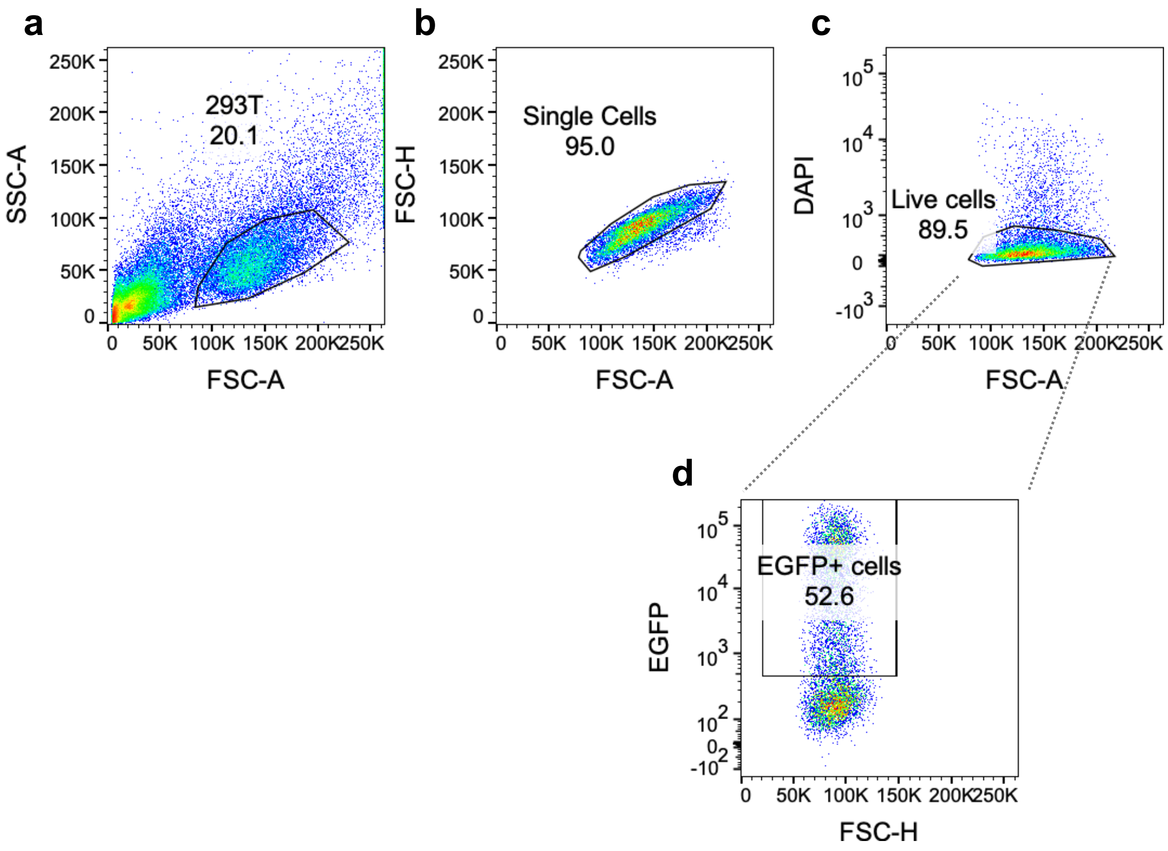

Supplementary Figure 7. Gating strategy of flow cytometery, related to Figs. 5f and 6f. **a-d** The transfected HEK 293T cells were gated into cells (**a**) by FSC- and SSC-scatter, followed by single cell selection (**b**) and dead cells exclusion (**c**), and then gated into EGFP+ cells (**d**) to measure its intensities. The figure was illustrated with HEK 293T cells transfected with WT EGFP- and mCherry-fused SLFN11-expression vectors.

Fig. 3b. Anti-mCherry

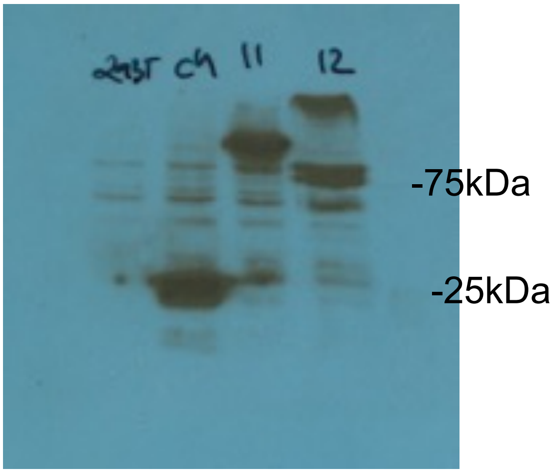

Fig. 3c. Anti-HIV-Gag-p24

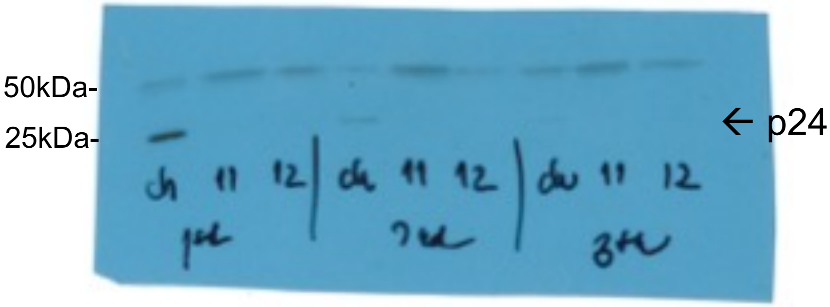

Supplementary Figure 8. Uncropped images of Western blots, related to Figs. 3b and 3c.
The upper image is a result of anti-mCherry blot shown in Fig. 3b. The lower image shows triplicate experiment of HIV-Gag-p24 detection from the transfected HEK293T cells. The first replicate (the left) was used in Fig. 3c. The lysates and supernatants from HEK 293T cells co-transfected with pNL-E plus pmCherry ("ch"; mock), pmCherry-SLFN11 ("11"), or pmCherry-SLFN12 ("12") were used for those experiments, respectively. "293T", untransfected HEK 293T.

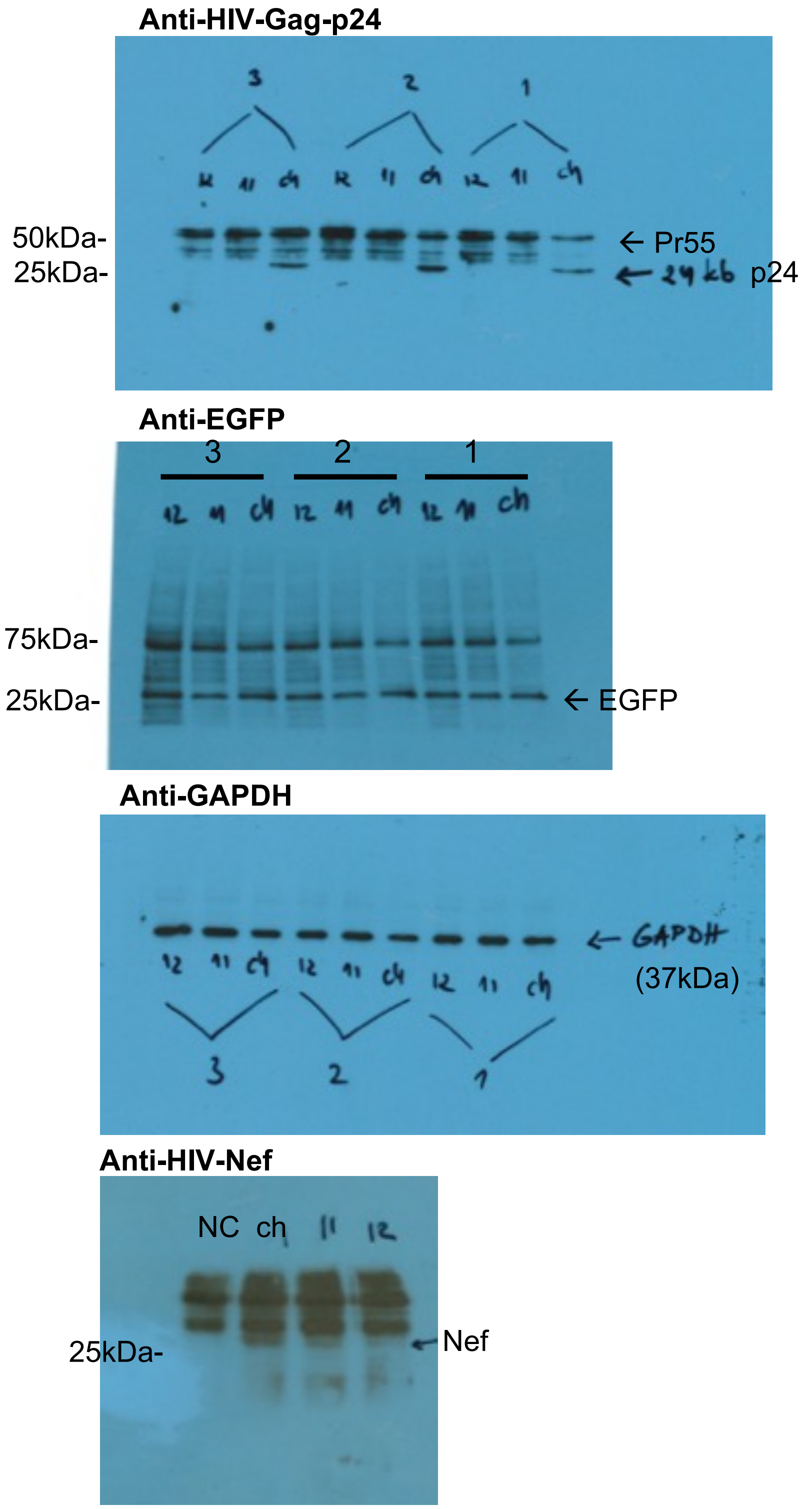

Supplementary Figure 9. Uncropped images of Western blots, related to Fig. 3g. Anti-HIV-Gag-p24, anti-EGFP and anti-GAPDH Western blot were performed with three independent samples (labelled with #1~3 in the images). The results from replicate #3 were used for the main figures. The bottom image shows a representative result of Western blot with anti-HIV-Nef antibody. The lysates from HEK 293T cells co-transfected with pNL-E plus pmCherry ("ch"; mock), pmCherry-SLFN11 ("11"), or pmCherry-SLFN12 ("12") were used. "NC", untransfected HEK 293T.

Anti-GAPDH

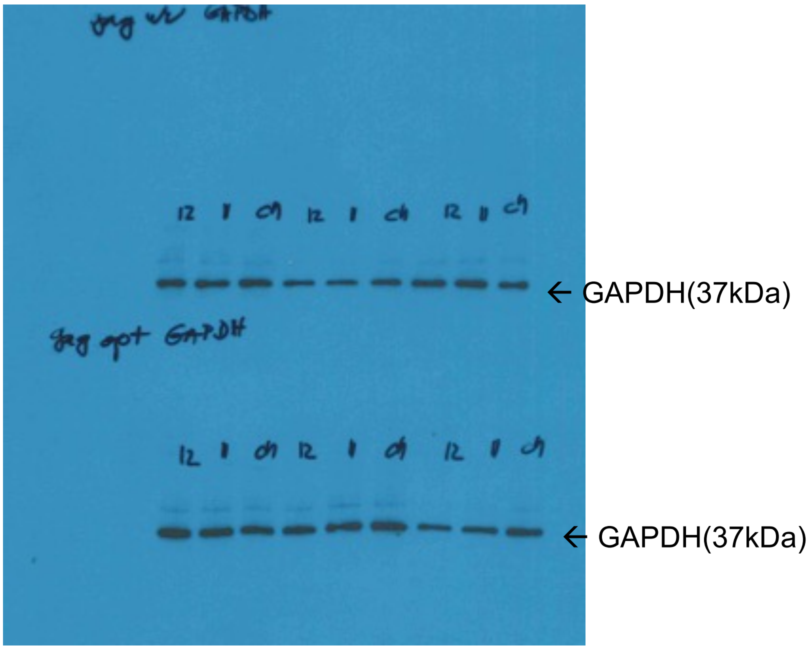

Anti-HIV-Gag-p24

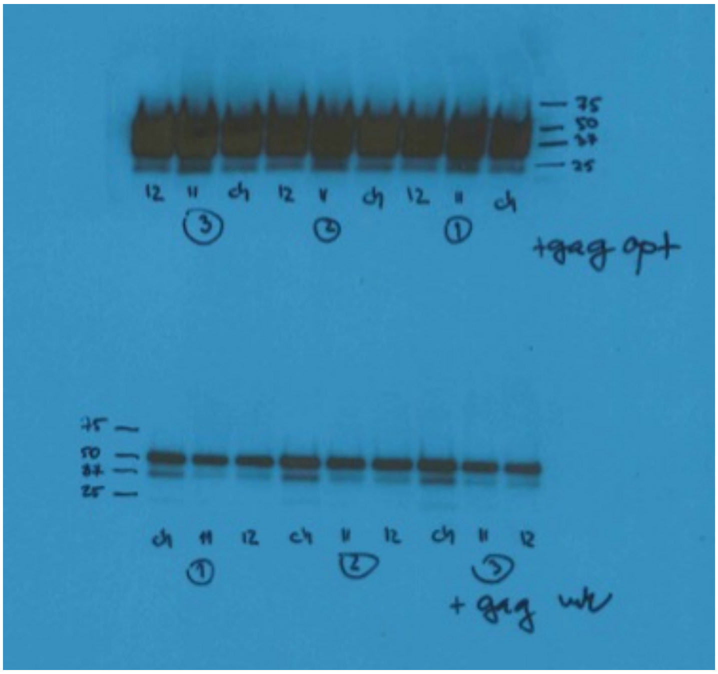

Supplementary Figure 10. Uncropped images of Western blots, related to Figs. 5c and 5d.

Upper image: The results of the anti-GAPDH Western blot with HEK 293T cells transfected with wild-type Gag (Gag-wt, upper slit) or codon-optimized Gag (Gag-opt, lower slit) expression vector and the indicated pmCherry vectors (“ch”, pmCherry alone; “11”, pmCherry-SLFN11; “12”, pmCherry-SLFN12). Lower image: the results of anti-HIV-Gag p24 Western blot with HEK 293T cells transfected with Gag-opt (upper slit) or Gag-wt (lower slit) expression vector, and the indicated pmCherry vectors.

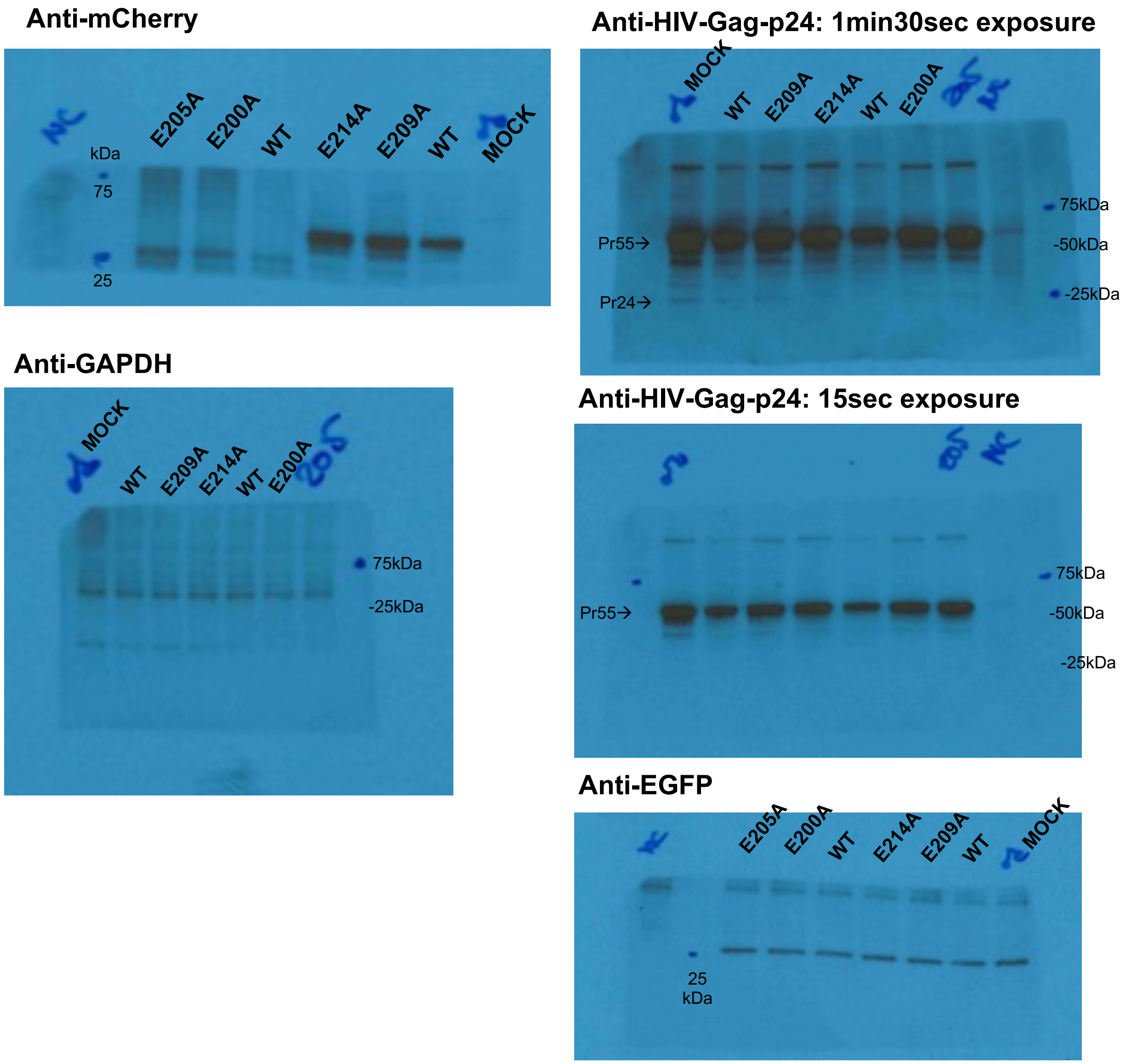

Supplementary Figure 11. Uncropped images of Western blots, related to Figs. 6d and 6e.

The top left panel shows an uncropped image of Fig. 6d. The rest four panels were images shown in Fig. 6e. The signals of anti-Gag-p24 Western blot by a 15-sec exposure was acquired for Gag Pr55 levels, while the image by a longer exposure (1.5 min) was used for p24 detection. Representative images of three independent experiments.

Supplementary Table 1. Clinical information of the patients used in Figs. 7a-c

Group	Median pVL (mini-max)	Median CD4 counts (min-max)
**HIV-High (n=16)**	12468 (50295-120000)	276.5 (11-726)
**HIV-Low (n=30)**	340 (25-10000)	672 (434-1343)
**VC (n=11)**	972 (55-1978)	588 (405-1840)
**EC (n=12)**	25 (25-50)	795 (245-1557)

pVL (patient viral load) : HIV-RNA copies/ml

Supplementary Reference, related to Fig. S2

1. Motulsky, H.J., Brown, R.E. (2006). Detecting outliers when fitting data with nonlinear regression – a new method based on robust nonlinear regression and the false discovery rate. BMC Bioinformatics 7, 123. 10.1186/1471-2105-7-123.
