# Supplementary material for: Schlafen 12 restricts HIV-1 latency reversal by a codon-usage dependent post-transcriptional block in CD4+ T cells
Source: Commun Biol. 2023 May 10;6:487. doi: 10.1038/s42003-023-04841-y (PMC10172343; doi:10.1038/s42003-023-04841-y)
Supplement: Supplementary file 3 — Description of Additional Supplementary Files [file 42003_2023_4841_MOESM3_ESM.pdf]

Description of Additional Supplementary Files

File name: Supplementary Data 1

Description: CPM values of each samples obtained from the RNA-seq

File name: Supplementary Data 2

Description: Gene list shown up by k-means clustering

File name: Supplementary Data 3

Description: List of known inhibitors of HIV-1 replication/function in the cluster I according to the NIH HIV interaction database

File name: Supplementary Data 4

Description: List of DEGs and members of gene families that contain a known restriction factor (RF)

File name: Supplementary Data 5

Description: List of the 58 candidate genes identified Fig. 1f

File name: Supplementary Data 6

Description: Raw data of relative synonymous codon usage (RSCU) shown in Fig. 5e

File name: Supplementary Data 7

Description: Oligonucleotides used in this study

File name: Supplementary Data 8

Description: Antibodies used in this study

File name: Supplementary Data 9

Description: Numerical source data related to Fig. 1d

File name: Supplementary Data 10

Description: Numerical source data related to Fig. 2

File name: Supplementary Data 11

Description: Numerical source data related to Fig. 3

File name: Supplementary Data 12

Description: Numerical source data related to Fig. 4

File name: Supplementary Data 13

Description: Numerical source data related to Fig. 5

File name: Supplementary Data 14

Description: Numerical source data related to Fig. 6

File name: Supplementary Data 15

Description: Numerical source data related to Fig. 7
