# Supplementary material for: Schlafen 12 restricts HIV-1 latency reversal by a codon-usage dependent post-transcriptional block in CD4+ T cells
Source: Commun Biol. 2023 May 10;6:487. doi: 10.1038/s42003-023-04841-y (PMC10172343; doi:10.1038/s42003-023-04841-y)
Supplement: Supplementary file 19 — Reporting Summary [file 42003_2023_4841_MOESM19_ESM.pdf]

nature portfolio

Corresponding author(s): Andreas Meyerhans, Juana Díez, Mie Kobayashi-Ishihara

Last updated by author(s): Apr 4, 2023

Reporting Summary

Nature Portfolio wishes to improve the reproducibility of the work that we publish. This form provides structure for consistency and transparency in reporting. For further information on Nature Portfolio policies, see our Editorial Policies and the Editorial Policy Checklist.

Statistics

For all statistical analyses, confirm that the following items are present in the figure legend, table legend, main text, or Methods section.

n/a	Confirmed	
☐	☒	The exact sample size (*n*) for each experimental group/condition, given as a discrete number and unit of measurement
☐	☒	A statement on whether measurements were taken from distinct samples or whether the same sample was measured repeatedly
☐	☒	The statistical test(s) used AND whether they are one- or two-sided<br>*Only common tests should be described solely by name; describe more complex techniques in the Methods section.*
☒	☐	A description of all covariates tested
☒	☐	A description of any assumptions or corrections, such as tests of normality and adjustment for multiple comparisons
☐	☒	A full description of the statistical parameters including central tendency (e.g. means) or other basic estimates (e.g. regression coefficient) AND variation (e.g. standard deviation) or associated estimates of uncertainty (e.g. confidence intervals)
☒	☐	For null hypothesis testing, the test statistic (e.g. *F*, *t*, *r*) with confidence intervals, effect sizes, degrees of freedom and *P* value noted<br>*Give P values as exact values whenever suitable.*
☒	☐	For Bayesian analysis, information on the choice of priors and Markov chain Monte Carlo settings
☒	☐	For hierarchical and complex designs, identification of the appropriate level for tests and full reporting of outcomes
☒	☐	Estimates of effect sizes (e.g. Cohen's *d*, Pearson's *r*), indicating how they were calculated

Our web collection on statistics for biologists contains articles on many of the points above.

Software and code

Policy information about availability of computer code

Data collection	FACS Diva software for the acquisition of flow cytometry data.
Data analysis	For the RNA-seq analyses, we described in the Methods section as follows:<br>"RNA-seq reads were mapped against the Homo sapiens reference genome (GRCh37.p13) with the GEMtools RNA-seq pipeline (http://gemtools.github.io/docs/rna_pipeline.html). Genes were quantified with the same pipeline using the Gencode version as an annotation. Normalization was performed with the edgeR TMM method. The k-means clustering was performed by iDEP. 94 interface and the heatmap was generated by ggplot2 R package. Gene ontology (GO) enrichment analysis was performed with DAVID (http://david.ncifcrf.gov/). Differentially expression analysis was performed with the 'robust' version of the edgeR R package. Genes with FDR<5% were considered differentially expressed. "<br><br>Graph Pad Prism7 was used for the rest of statistical analyses.<br><br>FlowJo ver.10 was used to analyze flow cytometry data.

For manuscripts utilizing custom algorithms or software that are central to the research but not yet described in published literature, software must be made available to editors and reviewers. We strongly encourage code deposition in a community repository (e.g. GitHub). See the Nature Portfolio guidelines for submitting code & software for further information.

Data

Policy information about availability of data

All manuscripts must include a data availability statement. This statement should provide the following information, where applicable:

- Accession codes, unique identifiers, or web links for publicly available datasets
- A description of any restrictions on data availability
- For clinical datasets or third party data, please ensure that the statement adheres to our policy

The RNA-seq data from this study are available from the NCBI Expression Omnibus GEO; GSE221332. The token is wtsnwymqvpmjbwj.

Human research participants

Policy information about studies involving human research participants and Sex and Gender in Research.

Reporting on sex and gender	For the RNA-seq analysis, we used the cultured naive CD4 + T cells isolated from 1 female and 2 male individuals. We validated the expressions of SLFN family genes using additional samples from 2 females and 3 males by RT-qPCR.<br><br>For the clinical analysis according to Fig. 6a-c, we used the following numbers of individuals:<br>HIV-High: 11 males and 5 females<br>HIV-Low: 15 males and 15 females<br>Elite controllers: 5 males and 7 females<br>Viremic controllers: 7 males and 4 females<br><br>For the FISH-flow analysis shown in Fig. 6d-f, we used samples from 1 female and 2 males.
Population characteristics	See clinical data section
Recruitment	Participants were recruited in Hospital Universitari Vall d'Hebron and IrsiCaixa in Cataluña, Spain.
Ethics oversight	Samples were obtained from the individuals with a written consent and by a protocol which was approved by the ethic committee in the Hospital Universitari Vall d'Hebron (PR(AG)270/2015), and IrsiCaixa (CEIC: EO-12-042 and PI-18-183).

Note that full information on the approval of the study protocol must also be provided in the manuscript.

Field-specific reporting

Please select the one below that is the best fit for your research. If you are not sure, read the appropriate sections before making your selection.

☒ Life sciences ☐ Behavioural & social sciences ☐ Ecological, evolutionary & environmental sciences

For a reference copy of the document with all sections, see nature.com/documents/nr-reporting-summary-flat.pdf

Life sciences study design

All studies must disclose on these points even when the disclosure is negative.

Sample size	Sample sizes were determined in line with the objectives of the statistical analysis. For the clinical samples, we determined the size as a compromise between adequate statistical power and limitations of the materials.
Data exclusions	Outlier values shown in Supplementary Fig. 2 were identified by ROUT method at maximum desired FDR (Q)=5% and excluded to calculate p-values.
Replication	Biological replicates were performed as specified in the Figure legend section.
Randomization	Randomization was not performed in this study.
Blinding	Patient names were only known by the clinicians but not given to laboratory scientists.

Reporting for specific materials, systems and methods

We require information from authors about some types of materials, experimental systems and methods used in many studies. Here, indicate whether each material, system or method listed is relevant to your study. If you are not sure if a list item applies to your research, read the appropriate section before selecting a response.

Materials & experimental systems

n/a	Involved in the study
☐	☒ Antibodies
☐	☒ Eukaryotic cell lines
☒	☐ Palaeontology and archaeology
☒	☐ Animals and other organisms
☐	☒ Clinical data
☒	☐ Dual use research of concern

Methods

n/a	Involved in the study
☒	☐ ChIP-seq
☐	☒ Flow cytometry
☒	☐ MRI-based neuroimaging

Antibodies

Antibodies used

anti-CD4 mAb; FITC labelled, anti-CD45RO mAb; ECD-labelled, anti-CD27 mAb; Alexa Fluor 700 labelled, anti-CD8 mAb;PE labelled, anti-mCherry mAb, anti-GAPDH mAb, anti-EGFP mAb, anti-HIV-1 Nef mAb, anti-HIV-1 p24 mAb, ECL anti-mouse IgG pAb, ECL anti-rabbit IgG pAb, Goat anti-Human IgG pAb, anti-SLFN12 mAb, anti-HIV-1 p24 mAb
The detailed product information is available in Supplementary Data 8.

Validation

All antibodies were commercially available and have been validated by the manufacturer.

Eukaryotic cell lines

Policy information about cell lines and Sex and Gender in Research

Cell line source(s)

HEK293T cell line was obtained from ATCC. ACH2 and TZM-bl cell lines were obtained from NIH AIDS Reagent Program. PBMCs for primary culture were obtained from 3 female- and 5 male-individuals as described above in the human research participants section.

Authentication

None of the cell lines used were authenticated.

Mycoplasma contamination

HEK293T cells were tested negative for mycoplasma contamination. The rest of the cell lines were not tested for the contamination.

Commonly misidentified lines
(See ICLAC register)

NA

Clinical data

Policy information about clinical studies
All manuscripts should comply with the ICMJE guidelines for publication of clinical research and a completed CONSORT checklist must be included with all submissions.

Clinical trial registration

No clinical trial in this study.

Study protocol

Not relevant to this study.

Data collection

Clinical data was collected from 69 HIV-1 infected individuals in IrsiCaixa, Badalona, Spain.

Outcomes

Classification of HIV-1 infected individuals was according to their HIV-1 RNA copies/mL (pVL) plus their history of anti-retroviral therapy (ART) in the past year. The criteria were as follows: (i) HIV-1 high viremic patients (HIV-High); >50.000pVL, (ii) HIV-1 low viremic patients (HIV-Low); <10.000pVL without ART, (iii) elite controllers (EC); <50pVL without ART, (iv) Viremic controllers (VC); <2000pVL without ART. The pVLs and CD4 counts of each patient group are summarized in Supplementary Table 1.

Flow Cytometry

Plots

Confirm that:

☒ The axis labels state the marker and fluorochrome used (e.g. CD4-FITC).

☒ The axis scales are clearly visible. Include numbers along axes only for bottom left plot of group (a 'group' is an analysis of identical markers).

☒ All plots are contour plots with outliers or pseudocolor plots.

☒ A numerical value for number of cells or percentage (with statistics) is provided.

Methodology

Sample preparation

Methods for the sample preparation were described in the Methods section of the manuscript.

Instrument	BD LSRFortessa (Becton, Dickinson)
Software	FACS Diva software for the acquisition of flow cytometry data and FlowJo v10 for the data analyses.
Cell population abundance	Not available.
Gating strategy	For generation of data for Fig. 1b, the cultured naive CD4 + T cells were gated according to size (FSC) and granularity (SSC). Doublets (FSC-A and FSC-H), dead cells (Live-dead aqua positive cells) and CD8 + cells (PE + cells) were excluded.<br><br>For acquiring GFP intensities in transfected HEK293T cells shown in Fig 5f and 6f, the cells were gated according to size (FSC) and granularity (SSC). Doublets (FSC-A and FSC-H) and dead cells (DAPI + cells) were excluded. The resulting viable single cells were gated into EGFP-positive cells to measure mean EGFP intensities.<br><br>Gating strategy for the FISH-flow analysis was described in Fig. 7d.

☒ Tick this box to confirm that a figure exemplifying the gating strategy is provided in the Supplementary Information.
