# Supplementary material for: Schlafen 12 restricts HIV-1 latency reversal by a codon-usage dependent post-transcriptional block in CD4+ T cells
Source: Commun Biol. 2023 May 10;6:487. doi: 10.1038/s42003-023-04841-y (PMC10172343; doi:10.1038/s42003-023-04841-y)
Supplement: Supplementary file 1 — Meyerhans_Peer Review File [file 42003_2023_4841_MOESM1_ESM.pdf]

nature portfolio

Peer Review File

Schlafen 12 restricts HIV-1 latency reversal by a codon-usage dependent post-transcriptional block in CD4+ T cells

Open Access This file is licensed under a Creative Commons Attribution 4.0 International License, which permits use, sharing, adaptation, distribution and reproduction in any medium or format, as long as you give appropriate credit to the original author(s) and the source, provide a link to the Creative Commons license, and indicate if changes were made. In the cases where the authors are anonymous, such as is the case for the reports of anonymous peer reviewers, author attribution should be to 'Anonymous Referee' followed by a clear attribution to the source work. The images or other third party material in this file are included in the article's Creative Commons license, unless indicated otherwise in a credit line to the material. If material is not included in the article's Creative Commons license and your intended use is not permitted by statutory regulation or exceeds the permitted use, you will need to obtain permission directly from the copyright holder. To view a copy of this license, visit http://creativecommons.org/licenses/by/4.0/.

Reviewers' comments:

Reviewer #1 (Remarks to the Author):

The manuscript by Kobayashi-Ishihara et al. describes the role of Schlafen 12 as an antiviral restriction factor that establishes a post-transcriptional block in HIV-1-infected cells. The topic is highly important and the data presented are well designed and controlled.
While the contribution of SLFN11 as a viral restriction factor was identified and proven recently, the influence of SLFN12 in viral defense mechanisms was incompletely understood. Although the cleavage of Leu UUA tRNA was shown very recently, the authors prove here that this cleavage mechanism by SLFN12 post-transcriptional inhibit HIV-1 replication and virus reactivation from latently infected cells. The authors demonstrate clearly that the inhibitory activity is dependent on HIV-1 specific codon usage and on the SLFN12 RNase activity.
The experiments described in this manuscript are well done, resulting in major advances in understanding the role of SLFN12 as viral restriction factor. I have no major concerns about the quality of the work presented in this manuscript except the structural part - please see below:
Major points:
1. The cryoEM structure of SLFN11 alone and in complex with tRNA was published recently Metzner et al. 2022. Please use the real structures in Figure 6a and b and not the predicted model. In Figure 6b a docking model is shown which is not well depicted and impossible to interpret. It is unclear from this figure to which domain the tRNA is docked and it is unintuitive that the tRNA is not docked to the positively charged cleft to the right. On top of that the cryo EM structure of human SLFN11 with tRNA has been ignored by the authors. Please use the structures available, optimize the figures and rewrite the text (page 12 starting from line 255).
It is unclear to me how the Uniplot (I guess Uniprot database) has been used to dock the tRNA to SLFN12. Please superimpose the SLFN11 tRNA cryo EM density (EMDB entry: EMD-14695) to the SLFN12 dimer to predict the tRNA interaction.

2. To Figure S5b: The result of the tRNA quantification in SLFN-transfected cells by RNA gel electrophoresis is not very clear. If one would use the 5S RNA for normalization the outcome would be different I guess. Maybe another staining procedure would increase the resolution?

Minor points:
Page 14 Line 281: the terminology is wrong here: residues E200 and E205 don`t belong to the cleavage site they are SLFN12 active site residues. The cleavage site is the position in the tRNA.

Reviewer #2 (Remarks to the Author):

In this manuscript, Mie Kobayashi-Ishihara et al., reported that SLFN12 is an HIV-1 restriction factor that establishes a post-transcriptional block, thereby inhibiting HIV-1 replication and virus reactivation from latently infected cells. This relies on the HIV-1 codon usage and on the RNase active sites in SLFN12. Within HIV-1-infected individuals, SLFN12 expression in PBMCs correlated with HIV-1 plasma viral loads and proviral loads. They also demonstrated that SLFN12 expression is enriched in infected cells positive for HIV-1 transcripts but not for HIV-1 proteins. They concluded that codon-usage dependent translation inhibition of HIV-1 proteins participates in HIV-1 latency and can restrict the amount of virus release after latency reversal. There are some major issues of experimental design as the initial screening of SLFN12 was from cell models without HIV infection and the subsequent biochemical and signaling studies in irrelevant models of HIV infection/latency in immune cells. Description of studies with patient cells are not clear and have many issues too. Therefore, it is not

cellar whether the discovery is truly relevant to HIV latency or latency reversal in HIV+ immune cells.
Major points:
1. In Figure 1, while it is great to have three models, mimic HSP and TCR-driven clone expression, no HIV infection was in these models. Considering the essential role of host-virus interaction in the signaling pathways for the evolvement of HIV replication and latency, this is the major concern for the study.
2. In Figure 2a, it is not clear whether there is any statistical difference of SLFN12 expression between HSP and TCR T cells. This are the important starting data to define the possible role of SLFN12 as RF.
3. Data in Figures 2-6 and biochemical studies were based on either ACH2 or HEK293T. This has to be studied in the primary CD4 T cell model of latency and/or primary CD4+ T cells, or validated in the primary CD4+ T cell models.
4. In Figure 7a-c, it is not clear whether they were from PBMCs or isolated CD4+ T cells. Also, in Figure 7d-f, it is not clear which cells were used, patient cells or HIV-infected CD4 T cells. If they are from patient cells, which group of patients they were from, VC, EC, high, or low, or mixed groups. If from mixed group, can the data answer the question whether CLFN12 is a true HIV RF? Were they on long-term ART or not? Were they statistically different? If SLFN12 reduces, what happens to HIV expression in these patient immune cells.

Reviewer #3 (Remarks to the Author):

In this manuscript, the authors identified SLFN12 as a restriction factor for HIV reactivation in latently infected T cells by comparing gene expression patterns in PBMC T cells cultured under homeostatic proliferation conditions (with IL-7 and IL-15) or TCR signaling activation conditions. In the ACH2 model of HIV latency, knock down of SLFN12 facilitated SAHA-induced HIV reactivation and Gag protein translation efficiency. Ectopic expression of SLFN11 or SLFN12 in HEK 293T cells suppressed expression of co-transfected HIV-1 provirus Gag protein but not Gag RNA, causing ribosome pausing on HIV-1 Gag mRNA, without affecting global mRNA translation. Optimization of the codon usage in the HIV Gag mRNA reversed this phenotype, allowing Gag protein expression in the presence of SLFN11 and SLFN12. Leu-UUA is enriched in the HIV genome compared to humans; substitution of leucine codons in EGFP with UUA caused expression of EGFP to be downregulated by co-expression of SLFN11 or SLFN12. Suppression of HIV virus titer was alleviated by mutation of the RNase active sites of SLFN12. Consistent with a role in maintenance of HIV latency, in PBMCs from HIV-infected individuals, SLFN12 expression was highest in cells expressing HIV-1 RNA but not p24 protein. The authors hypothesize that blocking SLFN11 and SLFN12 expression could induce reactivation of HIV virus protein production in latently-infected T cells, exposing the virally-infected cells to an immune response.

This is an exciting report, documenting an effect of SLFN12 on HIV protein production with possible consequences for eliminating latent infections in HIV-positive patients. The only substantial criticism is to properly cite and discuss relevant published work; in particular, that the physiological substrate of SLFN12 RNase activity is tRNA-Leu-TAA.

Specific Comments:
Fig 1b: The abbreviations in the figure should be explained in the figure legend

Fig 1h and Fig S2: Why does the SLFN11 expression pattern in T cells from the 5 additional donors in Fig S2 look different from the SLFN11 expression pattern in Fig 1h and instead look similar to that of SLFN12 in Fig 1h? The text says that the SLFN12 expression pattern “differed from the other SLFN family mRNAs”, although in Fig S2, SLFN 13 also looks similar to SLFN12.

Figure 2D and 2E: Knock down of SLFN11 also had a significant effect on SAHA-induced HIV reactivation and Gag protein translation in the ACH2 cells. The effect is therefore not specific for SLFN12. This point should be discussed.

Fig 3: Once again, no comparison is made between the effects of SLFN12 ectopic expression and the identical effects of SLFN11 ectopic expression in the 293T cells. Please discuss.

Fig 4: Please explain in more detail the changes made during codon optimization of HIV Gag.

Fig 5: In the Results text, please cite also the first solved structure of SLFN12 and the discovery of SLFN12 RNase activity (Garvie et al., Nat Comm, 2021). SLFN12 is also known to degrade tRNA-Leu-TAA (Lee et al., Nat Chem Biol, 2022), and this work should be cited and discussed appropriately, and this section should be rewritten to take into account this previously published data.

Fig 6e: Please define Gag Pr55 and how expression of this protein is significant in contrast to Gag p24.

Fig 7d: Please define all labels used in the figure legend.

Response to reviewers

We thank the reviewers for their thoughtful and thorough revision of our work. Their suggestions have been helpful to improve the manuscript. We have now addressed all the issues brought up and modified the manuscript, whenever possible, as recommended.

We hope the reviewers will find these changes and our response to the comments satisfactory.

Attached below is a point-by-point response to each of the reviewer's comments. For clarity, we have highlighted the text written by the reviewers in black, and our response in blue**.**

Reviewer #1:

The manuscript by Kobayashi-Ishihara et al. describes the role of Schlafen 12 as an antiviral restriction factor that establishes a post-transcriptional block in HIV-1-infected cells. The topic is highly important and the data presented are well designed and controlled. While the contribution of SLFN11 as a viral restriction factor was identified and proven recently, the influence of SLFN12 in viral defense mechanisms was incompletely understood. Although the cleavage of Leu UUA tRNA was shown very recently, the authors prove here that this cleavage mechanism by SLFN12 post-transcriptional inhibit HIV-1 replication and virus reactivation from latently infected cells. The authors demonstrate clearly that the inhibitory activity is dependent on HIV-1 specific codon usage and on the SLFN12 RNase activity.

The experiments described in this manuscript are well done, resulting in major advances in understanding the role of SLFN12 as viral restriction factor. I have no major concerns about the quality of the work presented in this manuscript except the structural part - please see below:

Major points:

1. The cryoEM structure of SLFN11 alone and in complex with tRNA was published recently Metzner et al. 2022.

Please use the real structures in Figure 6a and b and not the predicted model. In Figure 6b a docking model is shown which is not well depicted and impossible to interpret. It is unclear from this figure to which domain the tRNA is docked and it is unintuitive that the tRNA is not docked to the positively charged cleft to the right. On top of that the cryo EM structure of human SLFN11 with tRNA has been ignored by the authors. Please use the structures available, optimize the figures and rewrite the text (page 12 starting from line 255). It is unclear to me how the Uniplot (I guess Uniprot database) has been used to dock the tRNA to SLFN12. Please superimpose the SLFN11 tRNA cryo EM density (EMDB entry: EMD-14695) to the SLFN12 dimer to predict the tRNA interaction.

We thank the reviewer for his helpful comments. We have now substituted the structure of the AlphaFold model of SLFN11 by the EM structure in PDB, 7ZEL. However, the structure of SLFN11 with tRNA was also obtained by docking in the publication of Metzner et al. (Ref#63) and this was not available in the PDB. To obtain a similar structure of SLNF11 with tRNA we have applied a docking with the same structure of tRNA used by Metzner et al. (the type I $tRNA^{Phe}$ from yeast, with code 5AXM in PDB). Then, we have repeated the docking with the structures of SLNF12 (from code 7LRE in PDB) and SLNF13 (retrieved from Uniprot and modelled by AlphaFold (Ref#87, Varadi et al. 2022)). To obtain the dimer structure of SLNF13, we used a superimposition of the AlphaFold monomer structure of SLFN13 in the structure of SLFN11 with Matchmaker (using Chimera), first superimposing in chain A and next in chain B. The following figure shows the resulting surfaces of the complex structures (in green the surface of $tRNA^{Phe}$, in beige the structure of the dimer of SLFN11, in cyan for SLFN12 and in magenta for SLFN13).

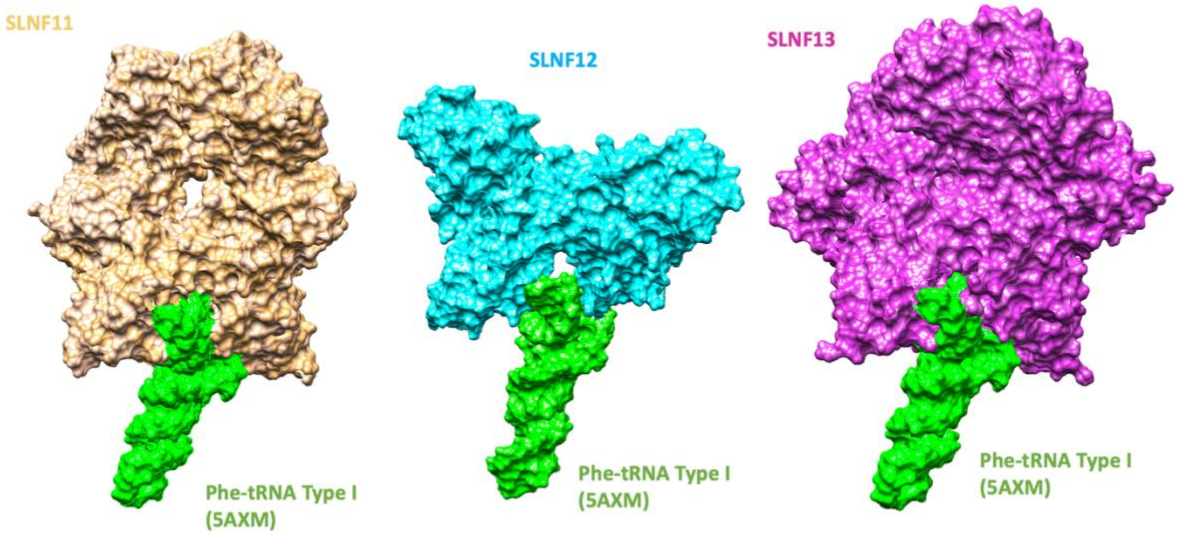

The structure of type II tRNA (selenocysteine tRNA, with code 3HL2 in PDB) was also docked, according to Yang et al. (Ref#49) for the model of SLFN13. See results in next figure, with the structure of Type II tRNA in yellow.

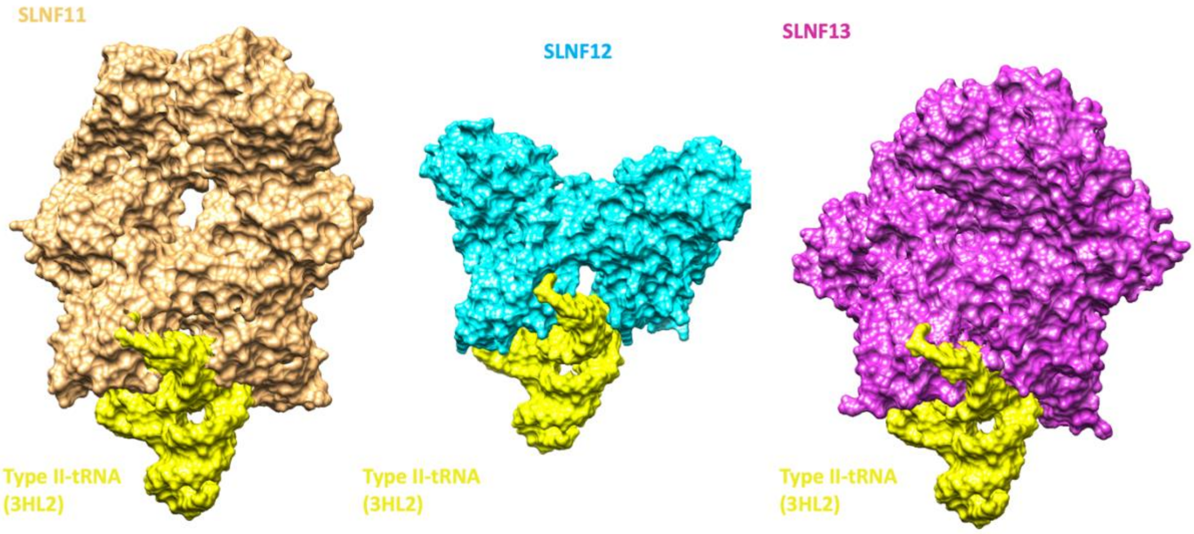

All complexes were further relaxed with Rosetta (Ref#89, Conway et al. 2014). We have used the structures of the dimer of SLFN12, docked and relaxed with the structures of $tRNA^{Phe}$ and selenocysteine tRNA, to show the active site and the role of Glutamates 200 and 205 in both chains of the dimer. We have modified figure 6 and its legend. The results in the main text of the manuscript are still valid, but we have also modified the methods section on "Structural comparison" (lines 541-551).

We have also modified the colors of figure 6. Certainly, the view of the Coulombic potential surface was unintuitive and didn't help to understand the location of the binding of tRNA. An additional view of the dimers of SLNF11 and SLNF12 help to understand that both SLFN11 and SLFN12 share large areas of positively charged potential, supporting the interaction with tRNAs. However, active binding and docking require the adequate surface complementarity which is reason to fit the tRNA in a cleft close to the active site, rather than in a more planar surface in the opposite site.

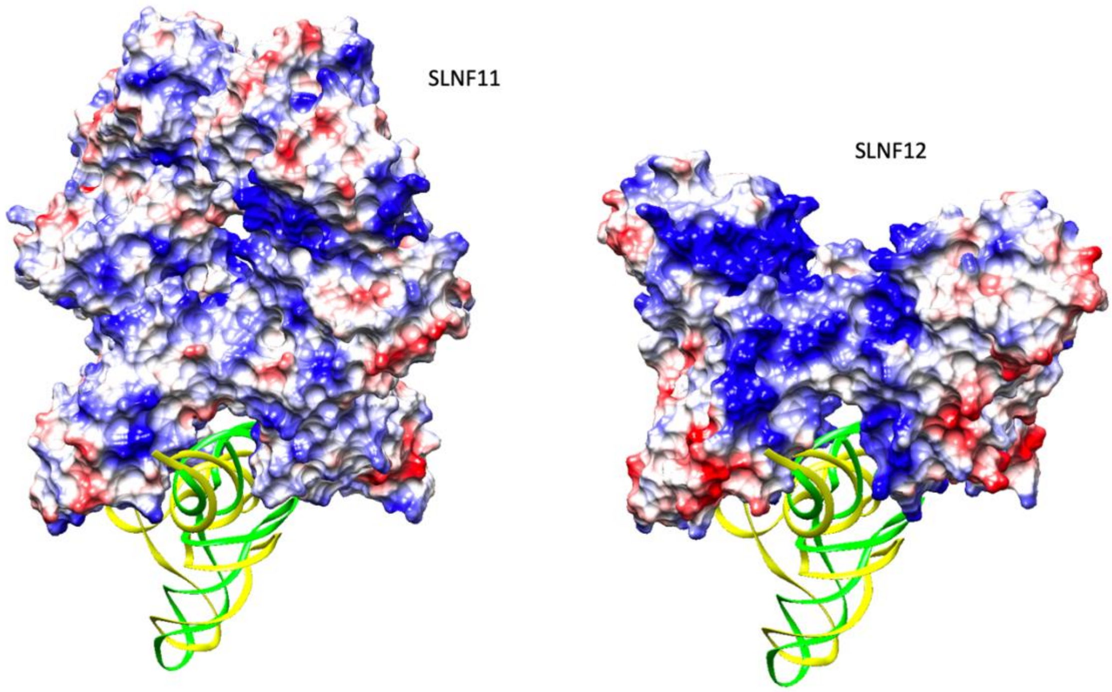

The revised Figs. 6a and b are as follows:

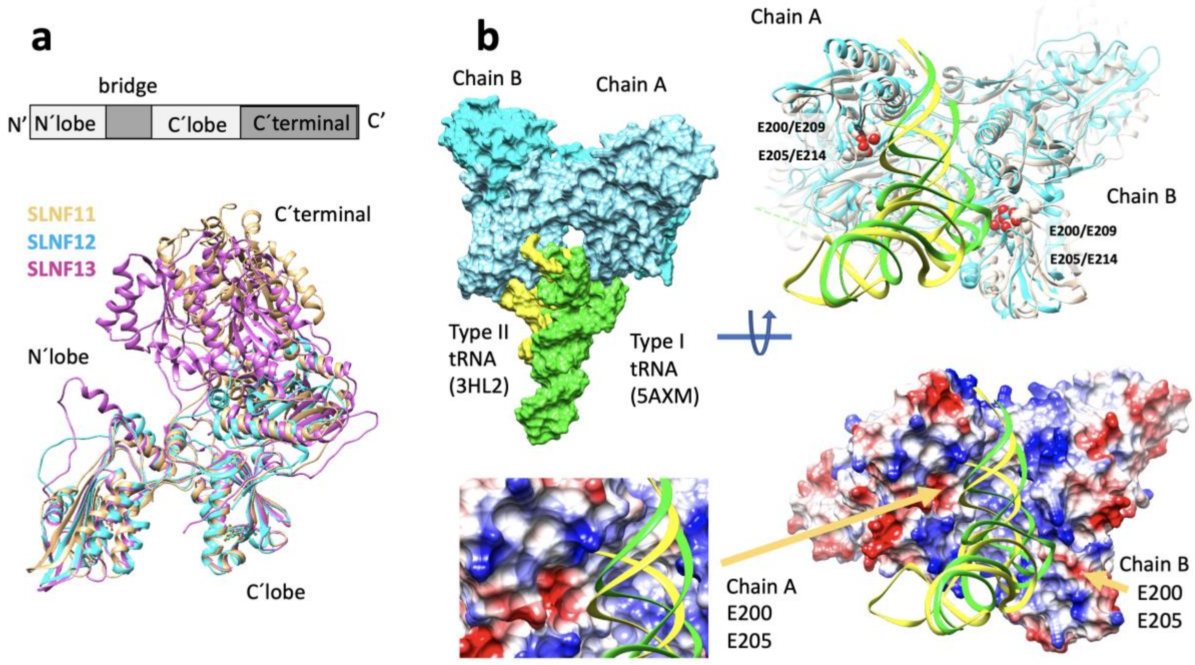

2. To Figure S5b: The result of the tRNA quantification in SLFN-transfected cells by RNA gel electrophoresis is not very clear. If one would use the 5S RNA for normalization the outcome would be different I guess. Maybe another staining procedure would increase the resolution?

We appreciate this comment. For tRNA quantification (Figure S5b) we have used as internal control 5.8S RNA as done by Li et al. (Ref#61). To test robustness, we now also normalized the values to 5S RNA as suggested and added the plot as Fig. S5c (see below). The trend of decrease in type II tRNA by SLFN12 appears similar and the conclusion is that type II tRNA was decreased by SLFN12 although less than by SLFN11. These data are in line with the recent observations of the specific cleavage of the low abundant Leu-UUA tRNA by SLFN12 (Ref#60, Lee et al 2022) and the broader cleavage activity of SLFN11 (Ref#61, Li et al. 2018, Ref#63 Metzner et al. 2022). Figure legend and text was modified accordingly (lines 292, 293, and 345~348).

Fig. S5c:

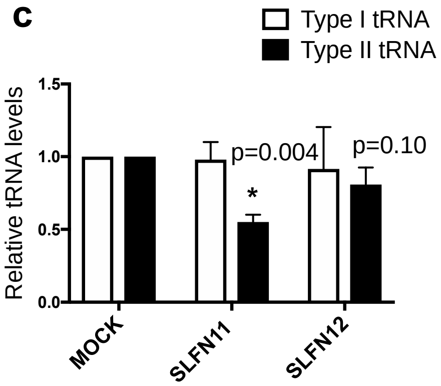

Minor points:
Page 14 Line 281: the terminology is wrong here: residues E200 and E205 don`t belong to the cleavage site they are SLFN12 active site residues. The cleavage site is the position in the tRNA.

We completely agree and now changed the terminology accordingly (line 286).

Reviewer #2:
In this manuscript, Mie Kobayashi-Ishihara et al., reported that SLFN12 is an HIV-1 restriction factor that establishes a post-transcriptional block, thereby inhibiting HIV-1 replication and virus reactivation from latently infected cells. This relies on the HIV-1 codon usage and on the RNase active sites in SLFN12. Within HIV-1-infected individuals, SLFN12 expression in PBMCs correlated with HIV-1 plasma viral loads and proviral loads. They also demonstrated that SLFN12 expression is enriched in infected cells positive for HIV-1 transcripts but not for HIV-1 proteins. They concluded that codon-usage dependent translation inhibition of HIV-1 proteins participates in HIV-1 latency and can restrict the amount of virus release after latency reversal. There are some major issues of experimental design as the initial screening of SLFN12 was from cell models without HIV infection and the subsequent biochemical and signaling studies in irrelevant models of HIV infection/latency in immune cells. Description of studies with patient cells are not clear and have many issues too. Therefore, it is not cellar whether the discovery is truly relevant to HIV latency or latency reversal in HIV+ immune cells.

Major points:
1. In Figure 1, while it is great to have three models, mimic HSP and TCR-driven clone expression, no HIV infection was in these models. Considering the essential role of host-virus interaction in the signaling pathways for the evolvement of HIV replication and latency, this is the major concern for the study.

The aim of the RNA-seq analysis of Fig.1 was to screen for candidate host restriction factors in CD4+ T cells with a culture condition that is refractory to HIV reactivation. From these experiments we identified SLFN12 as a candidate restriction factor and then verified it through the subsequent experiments. This is a valid scientific procedure.

Of note, to include a homogenous latently HIV-infected population of naïve T cells in these experiments is technically impossible. In our hands, the maximum percentage of latently infected cells that one can obtain was 2.6% (Ref#16, Tsunetsugu-Yokota et al. 2016).

2. In Figure 2a, it is not clear whether there is any statistical difference of SLFN12 expression between HSP and TCR T cells. This are the important starting data to define the possible role of SLFN12 as RF.

Differences in the expression of SLFN12 in HSP- and TCR-cultured T cells are now shown in Figures 1h and 2a. In both cases, the difference in expression was statistically significant (FDR = 4.53×10^{-5} and adjusted p = 0.0145 by ordinary one-way ANOVA, respectively). The first value was already given in Figure 1h. We have now added the missing p-value of Figure 2a into Figure S2. Accordingly, we modified the text in the figure legend (lines 966~967). In addition, we corrected the number of tested individuals in the legend of Figure 2a (line 963).

3. Data in Figures 2-6 and biochemical studies were based on either ACH2 or HEK293T. This has to be studied in the primary CD4 T cell model of latency and/or primary CD4+ T cells, or validated in the primary CD4+ T cell models.

As stated above, it is technically not possible to generate a homogenous population of HIV-1 latently infected naïve CD4 T cells and therefore, one needs cell line models to evaluate the function of SLFN12. ACH2 cells are a well-established model of HIV-1 latency. HEK293T are a standard cell line for studies of HIV-1 production. With these models we clearly demonstrate that reduction in SLFN12 leads to increase of HIV-1 protein production while expression of SLFN12 reduces it. Furthermore, we validated the potential inhibitory effect of SLFN12 in ART-treated patients by taking advantage of the single-cell analysis technique “FISH-flow” (Figs. 7d-f). Together the results clearly define SLFN12 as a HIV-1 restriction factor.

4. In Figure 7a-c, it is not clear whether they were from PBMCs or isolated CD4+ T cells. Also, in Figure 7d-f, it is not clear which cells were used, patient cells or HIV-infected CD4 T cells. If they are from patient cells, which group of patients they were from, VC, EC, high, or low, or mixed groups. If

from mixed group, can the data answer the question whether CLFN12 is a true HIV RF? Were they on long-term ART or not? Were they statistically different? If SLFN12 reduces, what happens to HIV expression in these patient immune cells.

For Figures 7a-c, PBMCs from ART-untreated patients (covering VC, EC, high and low viremic patients) were used as described in the methods section (lines 445~452) and the Figure legend (line 1095). For Figures 7d-f, CD4+ T cells from ART-treated patients were used as described in the method section (lines 558~578) and the figure legend (line 1106).

Being a viral restriction factor is a general feature of a protein and should per se not be restricted to specific patient groups. Our statement of SLFN12 as a HIV-1 restriction factor is based on the observations we collected throughout the present study, not only from these primary models. The FISH-flow analysis of immune cells from HIV-1 patients showed that SLFN12 was highest in cells expressing HIV RNA but not p24. Conversely, reduced SLFN12 expression was enriched in cells that express p24. These results support the putative role of SLFN12 in the control of HIV-1 latency. This point and the technical limitations in the FISH-flow assay are carefully addressed in the Discussion section (lines 371~382).

Reviewer #3:
In this manuscript, the authors identified SLFN12 as a restriction factor for HIV reactivation in latently infected T cells by comparing gene expression patterns in PBMC T cells cultured under homeostatic proliferation conditions (with IL-7 and IL-15) or TCR signaling activation conditions. In the ACH2 model of HIV latency, knock down of SLFN12 facilitated SAHA-induced HIV reactivation and Gag protein translation efficiency. Ectopic expression of SLFN11 or SLFN12 in HEK 293T cells suppressed expression of co-transfected HIV-1 provirus Gag protein but not Gag RNA, SLFN12 in HEK 293T cells suppressed expression of co-transfected HIV-1 provirus Gag protein but not Gag RNA, causing ribosome pausing on HIV-1 Gag mRNA, without affecting global mRNA translation. Optimization of the codon usage in the HIV Gag mRNA reversed this phenotype, allowing Gag protein expression in the presence of SLFN11 and SLFN12. Leu-UUA is enriched in the HIV genome compared to humans; substitution of leucine codons in EGFP with UUA caused expression of EGFP to be downregulated by co-expression of SLFN11 or SLFN12. Suppression of HIV virus titer was alleviated by mutation of the RNase active sites of SLFN12. Consistent with a role in maintenance of HIV latency, in PBMCs from HIV-infected individuals, SLFN12 expression was highest in cells expressing HIV-1 RNA but not p24 protein. The authors hypothesize that blocking SLFN11 and SLFN12 expression could induce reactivation of HIV virus protein production in latently-infected T cells, exposing the virally-infected cells to an immune response.
This is an exciting report, documenting an effect of SLFN12 on HIV protein production with possible consequences for eliminating latent infections in HIV-positive patients. The only substantial criticism is to properly cite and discuss relevant published work; in particular, that the physiological substrate of SLFN12 RNase activity is tRNA-Leu-TAA.

Specific Comments:
Fig 1b: The abbreviations in the figure should be explained in the figure legend.

We apologize for this neglect. The abbreviations are now defined in the figure legend (lines:938~940).

Fig 1h and Fig S2: Why does the SLFN11 expression pattern in T cells from the 5 additional donors in Fig S2 look different from the SLFN11 expression pattern in Fig 1h and instead look similar to that of SLFN12 in Fig 1h? The text says that the SLFN12 expression pattern "differed from the other SLFN family mRNAs", although in Fig S2, SLFN 13 also looks similar to SLFN12.

We appreciate the careful comparison of the figures. Please note that our primary goal in this manuscript was to identify a host factor that fulfils the cluster I expression profile as shown in Fig. 1c. From our transcriptome data (Fig.1h), only SLFN12 expression was close to this and thus we wrote "SLFN12 expression pattern differed from the other SLFN family mRNAs". The subsequent SLFN12 qPCR analysis from additional blood donors (Fig. S2) showed a consistent expression pattern. All other SLFNs (Figs. 1h and S2) did not show a trend towards a decrease from HSP+TCR to TCR conditions and thus were considered non-consistent with a cluster I pattern.

To better understand our line of thoughts for the difference of SLFN12 to the other SLFNs and facilitate comparison of the expression patterns between RNA-seq and RT-qPCR, we have now changed the plotting style of Fig. S2 and added several p-values on the plots. We also added a phrase at lines 169~171.

The variability between the transcriptome data of the 3 blood donors (Figs. 1h and S3) and the qPCR data from the additional 5 donors (Fig. S2) for all other SLFNs than SLFN12 is not entirely clear however may relate to large inter-individual SLFN expression differences and the strong dependence on the immune status of the host (see Fig. 7a). As this was not a relevant issue in our present work, we did not follow it up.

The revised Fig. S2:

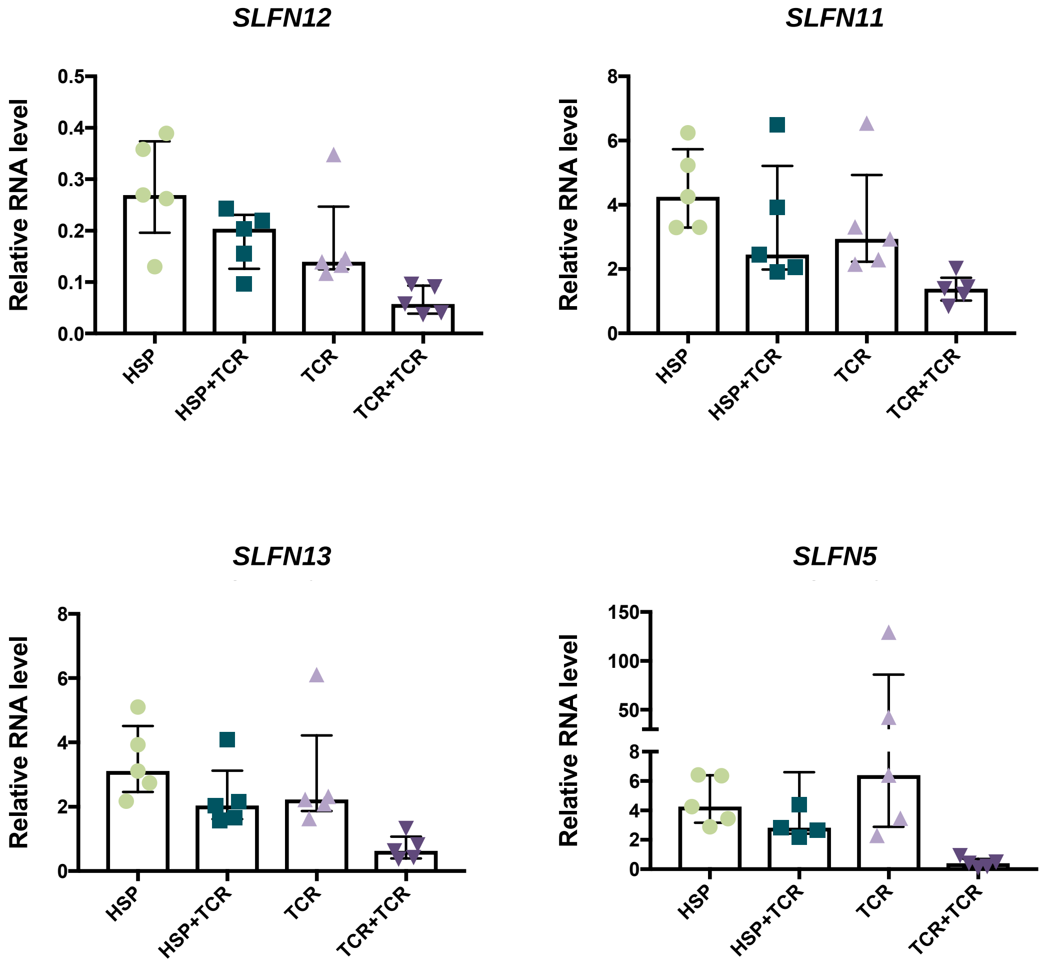

Figure 2D and 2E: Knock down of SLFN11 also had a significant effect on SAHA-induced HIV reactivation and Gag protein translation in the ACH2 cells. The effect is therefore not specific for SLFN12. This point should be discussed.

We totally agree with the reviewer. However, SLFN11 is known to have a translational effect on HIV-1 protein expression and was used as a positive control throughout our experiments with SLFN12. To make this point clearer to the reader, we now added a line on page 9, line 183 and added the SLFN11 results in the description of the experiments at page 10 (lines 194, 195 and 197). A line comparing SLFN11 and SLFN12 is also added in the discussion section (lines 345~348).

Fig 3: Once again, no comparison is made between the effects of SLFN12 ectopic expression and the identical effects of SLFN11 ectopic expression in the 293T cells. Please discuss.

As above, we now added SLFN11 to the description of the results on page 11, line 211.

Fig 4: Please explain in more detail the changes made during codon optimization of HIV Gag.

The codon-optimized gag sequence used in the present study has been obtained from the AIDS reagent program of the National Institute of Health (reference #8675). The respective plasmid has been constructed and provided to the reagent program by Drs. Yingying Li, Feng Gao and Beatrice H. Hahn. It was produced by swapping each HIV-1 gag codon into the most frequent synonymous codon. We

have now added the specific reference in the method section (lines 468~469), and added a phrase in the acknowledgement.

Fig 5: In the Results text, please cite also the first solved structure of SLFN12 and the discovery of SLFN12 RNase activity (Garvie et al., Nat Comm, 2021). SLFN12 is also known to degrade tRNA-Leu-TAA (Lee et al., Nat Chem Biol, 2022), and this work should be cited and discussed appropriately, and this section should be rewritten to take into account this previously published data.

We thank the reviewer for pointing this out. We have now added the respective references (#59 and 60) and changed the text in the results (lines 260~262 and 266~267) and discussion sections (lines 345~348 and 358).

Fig 6e: Please define Gag Pr55 and how expression of this protein is significant in contrast to Gag p24.

Gag Pr55 is the primary translated gag precursor protein from which the capsid Gag p24 is generated by proteolytic cleavage. The increase in the expression of Gag Pr55 and Gag p24 in HEK 293T cells with the mutated SLFN11 or SLFN12 proteins relative to the wild type proteins are comparable. We now clarified this in the result section (line 282).

Fig 7d: Please define all labels used in the figure legend.

We now added the definition of the labels to the figure legends (lines: 1108~1113).

REVIEWERS' COMMENTS:

Reviewer #1 (Remarks to the Author):

The author addressed all of my raised concerns to my satisfaction, and I recommend publishing this work.

Reviewer #2 (Remarks to the Author):

I thank the authors to address my concerns. However, the concerns were not directly addressed.
1). It is clear that the host factor interaction with HIV (genome/RNA/protein/particles) is essential for the true mechanism of HIV infection, latency, or restriction in both scenarios. In the presence of HIV infection, data/signaling from Figure 1 may or may not be relevant to the cells infected with HIV. There are primary HIV latency models available with HIV genome in each of the CD4+ T cell after sorting and expansion.
2). I hold my opinion that, without validation of the findings from 293T cells and ACH2 cells (cancer cell models), data may or may be not true in primary CD4+ T cells.
3). In Figure 7, while PBMCs were used, why SLFN12 was evaluated with CD4_ T cells in 7a, RNA copies in 7b and HIV DNA in 7c. In 7d-f, were the patients under suppressive ART, viral rebound, or other PWH? If they were not from ART-suppressed PWH, any claims of latency reversal restriction is not true.

Reviewer #3 (Remarks to the Author):

The reviewers have satisfied my concerns.

Our specific response to the comments of reviewer 2

Reviewer #2:
I thank the authors to address my concerns. However, the concerns were not directly addressed.

1). It is clear that the host factor interaction with HIV (genome/RNA/protein/particles) is essential for the true mechanism of HIV infection, latency, or restriction in both scenarios. In the presence of HIV infection, data/signaling from Figure 1 may or may not be relevant to the cells infected with HIV. There are primary HIV latency models available with HIV genome in each of the CD4+ T cell after sorting and expansion.

Our answer:
The experiments of figure 1 were designed after own observations in a primary HIV latency model of homeostatic proliferating cells. We found that under such homeostatic proliferating culture conditions, HIV remained latent despite stimulation via T cell receptors or SAHA treatment (Tsunetsugu-Yokota-et al., reference·16). The frequency of latently infected cells could never be expanded beyond 2.6%. Homeostatic proliferation conditions per se prevent efficient cell expansion and thus, it is not possible to obtain a homogenous population of latently infected cells. Aims of the experiments of figure 1 were to identify candidate restriction factors of HIV-1 and then later verify them by additional experiments. This is what we have done in our manuscript. It is a valid scientific procedure.

2). I hold my opinion that, without validation of the findings from 293T cells and ACH2 cells (cancer cell models), data may or may be not true in primary CD4+ T cells.

Our answer:
293T cells and ACH2 cells are commonly used for defining specific virus-host factor interactions. Our experiments of figures 2 – 6 then clearly demonstrate that SLFN12, like SLFN11 (a previously defined HIV-1 restriction factor; reference 45), is an HIV-1 restriction factor that acts at the level of translation. To demonstrate in-vivo-relevance, we have performed FISH-Flow experiments (figures 7d-f). Nonetheless, due to the technical limitations of that technique, there remain several unknows that we now specify in an **added paragraph** “limitation of the study” after the discussion (lines: 396~399). It is as follows:

Limitation of the study: The physiological conditions under which SLFN11 and/or SLFN12 restrict HIV-1 expression and suppress latency reversal *in vivo* are not completely defined. This includes infected cell types e.g. CD4 T cells and macrophages, location of function e.g. nuclear or cytoplasmic, and the activation of the RNase activity of the SLFNs.

3). In Figure 7, while PBMCs were used, why SLFN12 was evaluated with CD4_ T cells in 7a, RNA copies in 7b and HIV DNA in 7c. In 7d-f, were the patients under suppressive ART, viral rebound, or other PWH? If they were not from ART-suppressed PWH, any claims of latency reversal restriction is not true.

Our answer:
Experiments of figures 7a-c were performed to understand which conditions affect SLFN12 expression in PBMCs of HIV-1-infected individuals. SLFN12 transcript levels shown are normalized to TBP RNA levels (a house-keeping gene) and CD4 T cell counts that is variable amongst the patient groups (see Supplementary Table 1). The 3 figures demonstrate that the level of SLFN12 is increased by high virus load or proviral load. The higher the virus load, the

higher is immune system activation. As stated in the text (lines 364-367), data of figures 7a-c are consistent with a dependence of SLFN12 expression on the general activation of the immune system, and with SLFN genes being interferon inducible.

Concerning the patients of figures 7d-f, please check our previous response:
“For Figures 7d-f, CD4+ T cells from **ART-treated patients** were used as described in the method section (lines 564~570).
To facilitate patient data access, we have now **added information** about the treatment status of the patients into the legend of figure 7 (line 1122, “PBMCs from HIV-1-infected individuals without ART”; lines 1132-1133, “CD4+ T cells from patients on suppressive ART”).
